# The complete mitochondrial genome of *Gobio huanghensis* (Cypriniformes: Cyprinidae) in the Yellow River, China

**DOI:** 10.1080/23802359.2024.2318401

**Published:** 2024-02-22

**Authors:** Yanyan Du, Zijun Su, Zhuoyu Yang, Yanping Zhang, Zhongyu Lou, Shunwen Yang, Tai Wang

**Affiliations:** Gansu Fishery Resources and Environment in the Upper Reaches of the Yellow River Observation and Research Station, Gansu Fisheries Research Institute, Lanzhou, China

**Keywords:** Endemic species, gudgeon, mitogenome, phylogenetic relationship

## Abstract

*Gobio huanghensis*, a member of the eponymous genus within the Cyprinidae, family of the Cypriniformes order, is an endemic fish species found exclusively in the upper reaches of the Yellow River, spanning from Yinchuan to Lanzhou. This study presents the first comprehensive report of the complete mitochondrial genome sequence of *G. huanghensis*, encompassing 16,604 base pairs (bp) with a nucleotide composition of 26.3% cytosine (C), 17.6% guanine (G), 29.4% adenine (A), and 26.7% thymine (T). In congruence with other species in the *Gobio* genus, its mitochondrial genome comprises 37 genes, including two ribosomal RNA genes, 13 protein-coding genes (PCGs), and 22 transfer RNA genes. Notably, *COX1* initiates with the start codon GTG, distinct from the typical ATG start codon of other PCGs. The mitogenome exhibits four types of stop codons: TAA, TAG, TA-, and T--. Phylogenetic analyses, grounded in complete mitochondrial sequences, position *G. huanghensis at* the forefront of one of two major clusters within the genus *Gobio*, corroborating existing morphological classifications. These findings offer valuable theoretical insights for the taxonomic classification, conservation, and population genetics of *G. huanghensis.*

## 1. Introduction

*Gobio huanghensis* (Lo, Yao and Chen, 1977), a species within the genus *Gobio*, and subfamily Gobioninae (Teleostei: Cypriniformes: Cyprinidae), is native exclusively to the upper reaches of the Yellow River (Huang He) in China (Wang 1991). This species inhabits the river stretch between Yinchuan and Lanzhou, primarily feeding on benthic organisms*. G. huanghensis* typically spawns in May and June in areas characterized by gravelly bottoms and gentle currents (Wang 1991). However, due to the adverse impacts of human activities, the population of *G. huanghensis* has witnessed a significant decline (Qi and Yang 2009), leading to its inclusion in the China Species Red List (Wang and Xie 2009). Previous studies on *G. huanghensis* primarily centered around resource utilization, geographical distribution, and various biological and ecological aspects (Wu and Wu 1991; Chen 1998). While Qi and Yang (2009) provided *CYTB* sequences, comprehensive molecular and genetic data for the species remain sparse. In this study, we report the sequencing of the complete mitochondrial genome of *G. huanghensis* for the first time. This effort aims to furnish essential data for advanced genetic studies, biodiversity monitoring, and the conservation of this species.

## 2. Materials

**Figure 1.**
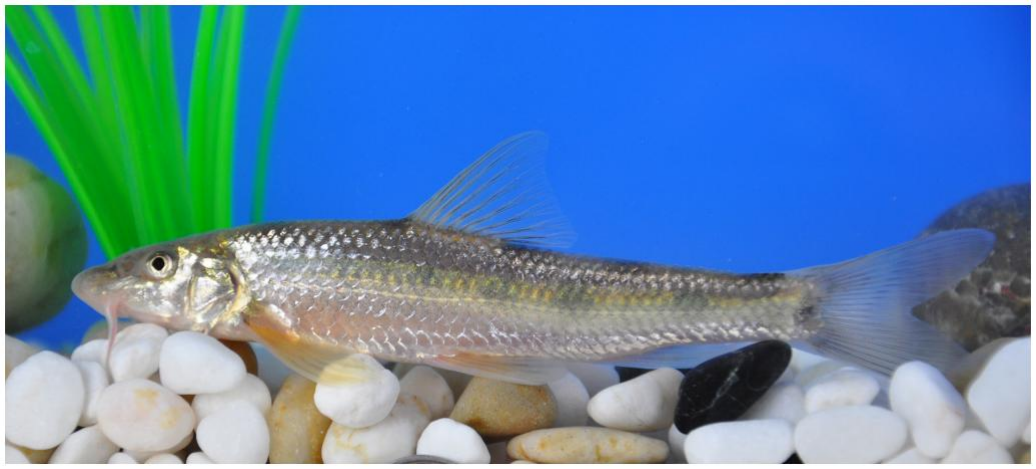
*G. huanghensis.* Specimen was taken from the Jingyuan County, Baiyin City, Gansu Province, China. Photographed by Tai Wang on 9 June 2021.

A specimen of *G. huanghensis* (Figure 1) was collected from Jingyuan County, Baiyin City, Gansu Province, China, at coordinates 104°37′48.63″E, 36°34′6.14″N, utilizing a cage net. Morphological identification was conducted in accordance with methodologies established by Wang (1991) and Chen (1998). *G. huanghensis* can be differentiated from other species in the *Gobio* genus based on several distinct characteristics: (1) the anus is situated midway between the base of the pelvic fin and the onset of the anal fin. (2) The species possess small eyes, with the head length exceeding 6.5 times the diameter of the eye. (3) The barbels are notably thick and elongated, extending rearward beyond the posterior margin of the anterior opercular bone. The collected specimen has been preserved and cataloged in the specimen room of the Gansu Fisheries Research Institute, Lanzhou, China, under the voucher number GSSCS-2021-0058 (Tai Wang, aqhongqi@qq.com).

## 3. Methods

Genomic DNA was isolated from muscle tissue using the Magnetic Animal Tissue Genomic DNA Kit (Xu et al. 2018), provided by Tiangen Biochemical Technology (Beijing) Co., Ltd. (Beijing, China). The concentration and purity of the nucleic acids were assessed using a NanoDROP 8000 ultramicro spectrophotometer. Primers were designed referencing the genomes of *Rhinogobio cylindricus* (KU379652) and *R. ventralis* (KU379653), and synthesized through OPC purification, with details available in Supplementary Material S1. The mitochondrial genome was amplified via polymerase chain reaction (PCR) using a 30 μL Taq PCR Master Mix. This reaction included genomic DNA at 0.67 ng/μL, primers at 0.33 nM, dNTPs at 0.2 mM, MgCl_2_ at 1.5 mM, and Taq polymerase at 0.05 U/μL. The PCR protocol involved initial denaturation at 95 °C for 5 min, followed by 35 cycles of denaturation at 95 °C for 30 s, annealing at 60 °C for 30 s, and extension at 72 °C for 1 min, concluding with a final elongation at 72 °C for 5 min. Agarose gel electrophoresis of the 16 primer pairs is detailed in Supplementary Material S2. The amplified PCR products were sequenced using the ABI 3730xl DNA Analyzer from Applied Biosystems, Inc. (Foster City, CA). The segmented sequences obtained are listed in Supplementary Material S3. Sequence assembly was conducted using SeqMan (DNASTAR Inc., Madison, WI), with manual corrections applied for sequencing errors (Meyer 1994). Overlap sizes and positions are included in Supplementary Material S4. Annotation of the mitochondrial genome and a map of the mitogenome were performed using the MITOS WebServer (Bernt et al. 2013). Using Blast, we identified eight complete mitochondrial sequences from the *Gobio* genus in the NCBI database, each with an identity exceeding 91.8% (Table 1). *Gnathopogon herzensteini* and *G. imberbis*, members of the subfamily Gobioninae, were selected as outgroups. Mitogenome sequences of 10 species were aligned using ClustalW in MEGA 11 (Kumar et al. 2018). Phylogenetic analysis was carried out using MEGA11 to construct a maximum-likelihood (ML) phylogenetic tree with 1000 bootstrap replicates.

**Table 1.** Names, GenBank accession numbers, and references of 10 fish mitogenomes used in phylogeny reconstruction.

Species	Accession number	Reference
*Gnathopogon herzensteini*	MT295103	–
*Gnathopogon imberbis*	KR010927	Gao et al. (2015)
*Gobio coriparoides*	MN864250	Ge et al. (2020)
*Gobio rivuloides*	OP354223	–
*Gobio cynocephalus*	KU314700	Li et al. (2018)
***Gobio huanghensis***	**OM302528**	–
*Gobio macrocephalus*	MT632636	Yi and Fu (2020)
*Gobio soldatovi*	OP359052	–
*Gobio gobio*	AB239596	Saitoh et al. (2006)
*Gobio acutipinnatus*	MT632635	Yi and Fu (2020)

## 4. Results

**Figure 2.**
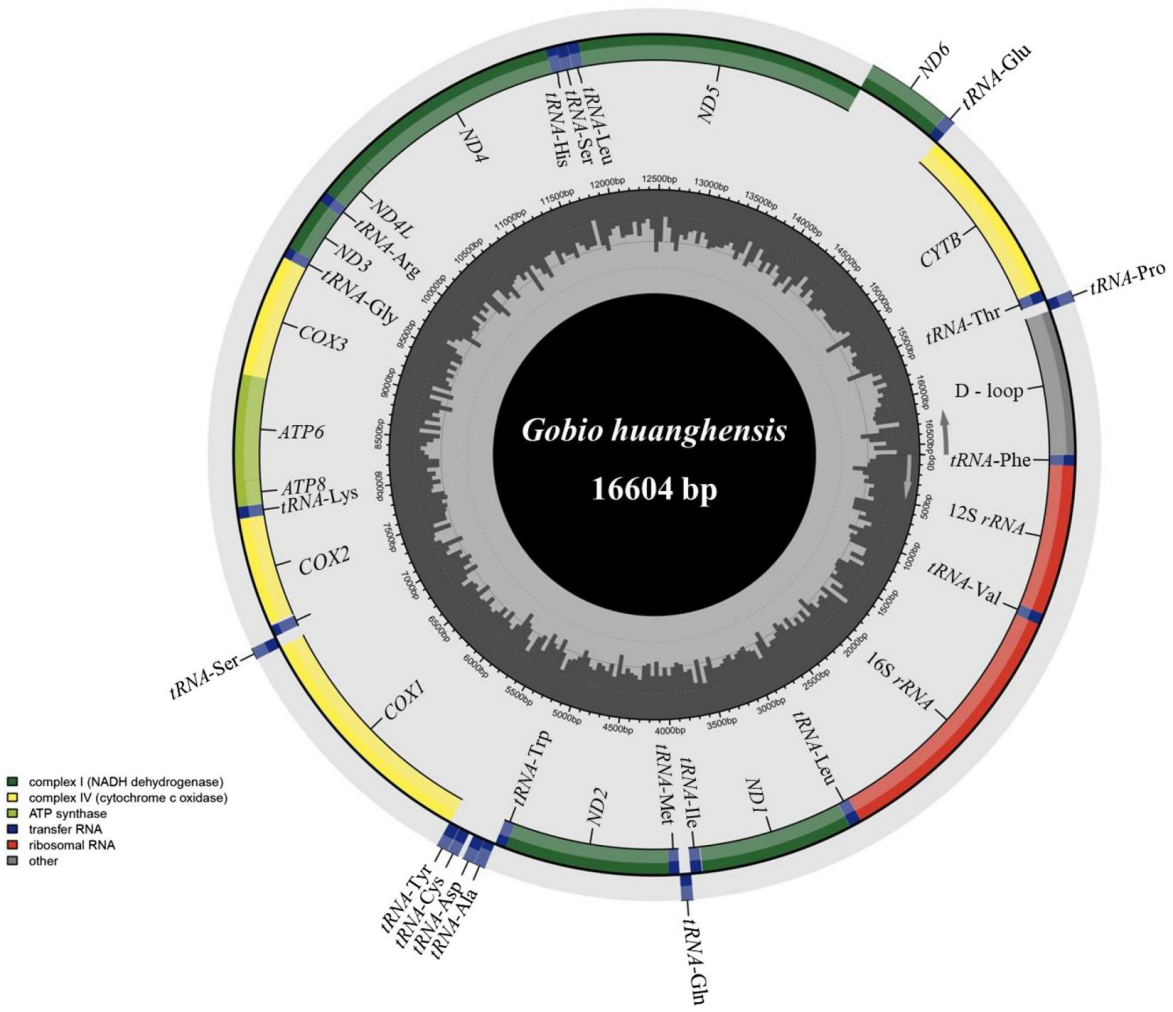
Complete mitochondrial genome map of *G. huanghensis* (GenBank accession no. OM302528).

**Figure 3.**
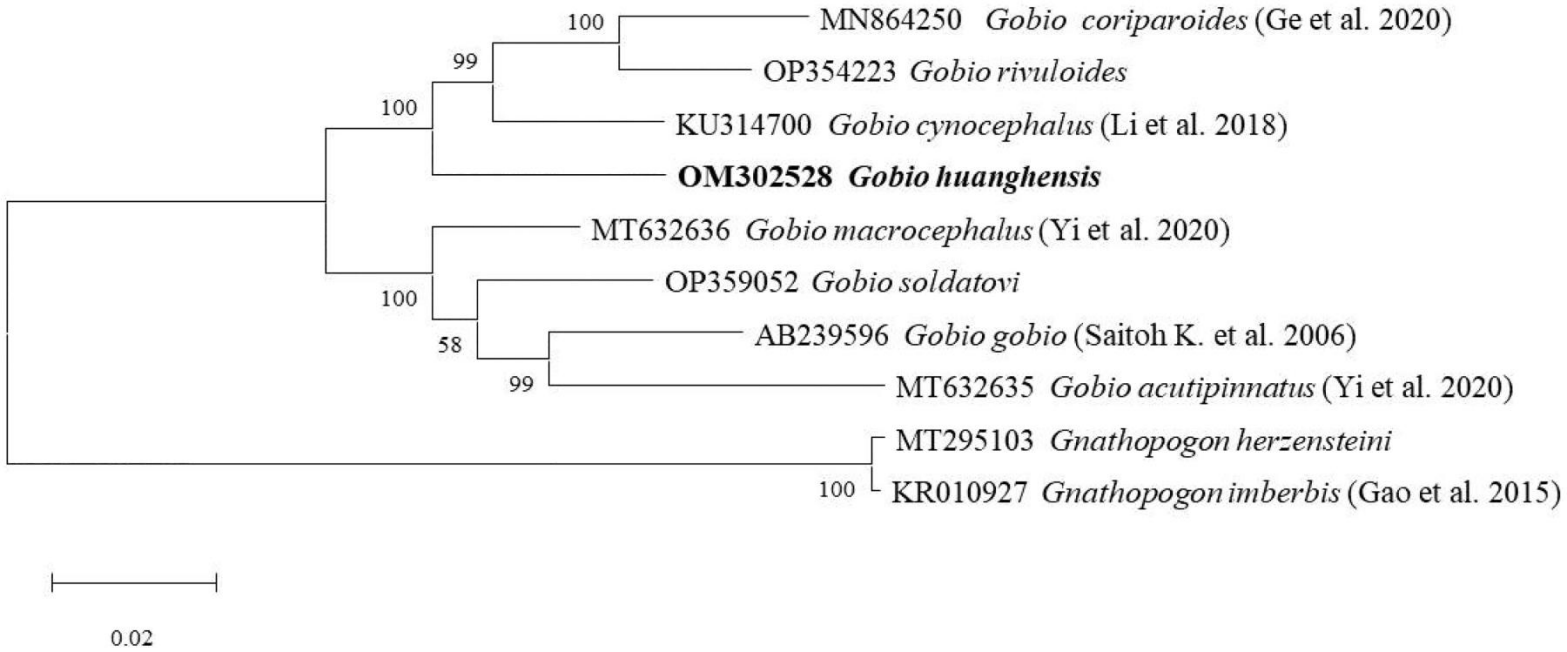
ML phylogenetic tree reconstructed using the mitochondrial genomes of eight *Gobio* fish species. The tree topology was evaluated with 1000 bootstrap replicates. Bootstrap values at the nodes correspond to the support values for the ML methods. A scale of 0.02 indicates genetic variation in the mitochondrial genome, equating to two differences per 100 nucleotides.

The complete mitochondrial genome sequence of *G. huanghensis* spans 16,604 base pairs (bp) and has been submitted to the GenBank database under the accession number OM302528. This mitochondrial genome comprises 13 protein-coding genes (PCGs: *ND1*–*ND6* and *ND4L*, *COX1*–*COX3*, *CYTB*, *ATP6*, and *ATP8*), two ribosomal RNA genes (16S *rRNA* and 12S *rRNA*), 22 transfer RNA genes (*tRNAs*), and a control region (Figure 2). The nucleotide composition is characterized by 29.4% adenine (A), 26.7% thymine (T), 26.3% cytosine (C), and 17.6% guanine (G). In the case of *G. huanghensis*, all PCGs initiate with the ATG start codon, with the exception of *COX1*, which starts with GTG. There are four types of stop codons employed: TAA serves as the stop codon for *COX1*, *ATP8*, *ND4L*, *ND5*, and *ND6*; TAG for *ND1*; T-- for *ND2*, *COX2*, *COX3*, and *CYTB*; and TA- for *ATP6*, *ND3*, and *ND4*. As shown in Figure 3, the mitochondrial genome of *Gobio* species forms two distinct sister clades.

Genes encoded on the L-strand and H-strand are shown outside and inside the circle, respectively. The arrows represent direction of transcription. The inner circle represents the GC content.

## 5. Discussion and conclusions

This study successfully presents the first complete assembly and annotation of the mitochondrial genome for *G. huanghensis*. The gene composition and arrangement in the mitochondrial genome of *G. huanghensis* are found to be consistent with those observed in other *Gobio* species’ mitogenomes (Ge et al. 2020; Yi and Fu 2020). Phylogenetic analysis using ML, based on the complete mitochondrial genomes of *G. huanghensis* and seven additional *Gobio* species, reveals that *G. huanghensis* forms a sister-group relationship with *G. cynocephalus*, *G. rivuloides*, and *G. coriparoides*. The mitochondrial genomic data acquired from *G. huanghensis* in this study will be instrumental in providing a genetic foundation for DNA barcoding, population genetics studies, and the conservation efforts of this endemic species.

## Supplementary Material

Supplemental Material

## Data Availability

The genome sequence data that support the findings of this study are openly available in GenBank of NCBI at https://www.ncbi.nlm.nih.gov/ under the accession no. OM302528. The associated ‘BioProject’ and ‘Bio-Sample’ numbers are PRJNA908272 and SAMN32034270.

## References

[CIT0001] Bernt M, Donath A, Jühling F, Externbrink F, Florentz C, Fritzsch G, Pütz J, Middendorf M, Stadler PF. 2013. MITOS: improved de novo metazoan mitochondrial genome annotation. Mol Phylogenet Evol. 69(2):313–319. doi:10.1016/j.ympev.2012.08.023. 22982435

[CIT0002] Chen YY. 1998. Cypriniformes II, Osteichthyes, Fauna Sinica. Beijing: Science Press.

[CIT0003] Gao TH, Tian HW, Wang DQ, Duan XB, Liu SP, Chen DQ. 2015. The mitochondrial genome of *Gnathopogon imberbis* (Cypriniformes, Cyprinidae). Mitochondrial DNA A DNA Mapp Seq Anal. 27(4):2543–2544. doi:10.3109/19401736.2015.1038796. 26017045

[CIT0004] Ge YS, Cheng QQ, Yan YB, Duan XC, Wang T, Zhang YP, Lou ZY, Du YY. 2020. The complete mitochondrial genome sequence and phylogenetic position of *Gobio coripa*roides. Mitochondrial DNA B Resour. 5(1):808–809. doi:10.1080/23802359.2020.1715864. 33366761 PMC7748591

[CIT0005] Tamura K, Stecher G, Kumar S. 2018. Mega11: molecular evolutionary genetic analysis version 11. Mol Biol Evol. 38(7):3022–3027. 10.1093/molbev/msab120 PMC8233496 33892491

[CIT0006] Li YH, Cao K, Fu CZ. 2018. Ten fish mitogenomes of the tribe *Gobionini* (Cypriniformes: Cyprinidae: Gobioninae). Mitochondrial DNA B Resour. 3(2):803–804. doi:10.1080/23802359.2018.1467236. 33490537 PMC7800260

[CIT0007] Meyer A. 1994. DNA technology and phylogeny of fish. In: Beaumont AR, editor. Genetics and evolution of aquatic organisms. Chapman and Hall; p. 219–249.

[CIT0008] Qi DL, Yang C. 2009. Cloning of mitochondrial cytochrome *b* gene of *Gobio huanghensis* and its phylogenetic relationships in genus *Gobio*. J Qinghai Univ (Nat Sci). 27(3):38–42.

[CIT0009] Saitoh K, Sado T, Mayden RL, Hanzawa N, Nakamura K, Nishida M, Miya M. 2006. Mitogenomic evolution and interrelationships of the cypriniformes (Actinopterygii: Ostariophysi): the first evidence toward resolution of higher-level relationships of the world’s largest freshwater fish clade based on 59 whole mitogenome sequences. J Mol Evol. 63(6):826–841. doi:10.1007/s00239-005-0293-y. 17086453

[CIT0010] Wang S, Xie Y. 2009. China species red list. Beijing: Higher Education Press.

[CIT0011] Wang XT. 1991. Vertebrate Fauna of Gansu. Lanzhou: Gansu Science and Technology Press.

[CIT0012] Wu YF, Wu CZ. 1991. The fishes of the Qinghai·Xizang Plateau. Chengdu: Sichuan Science and Technology Press.

[CIT0013] Xu P, Zhang CH, Sun ZY, Yu L, Feng K. 2018. Rapid extraction of genomic DNA by magnetic beads method. Chin J Bioinformatics. 16(3):190–195.

[CIT0014] Yi TY, Fu CZ. 2020. Two mitochondrial genomes of freshwater gudgeons in the genus *Gobio* (Cypriniformes: Gobionidae). Mitochondrial DNA B Resour. 5(3):3054–3055. doi:10.1080/23802359.2020.1797569. 33458056 PMC7782080

