# Supplementary material for: The complete mitochondrial genome of Gobio huanghensis (Cypriniformes: Cyprinidae) in the Yellow River, China
Source: Mitochondrial DNA B Resour. 2024 Feb 22;9(2):300–3. doi: 10.1080/23802359.2024.2318401 (PMC10885765; doi:10.1080/23802359.2024.2318401)
Supplement: Supplemental Material [file TMDN_A_2318401_SM4450.pdf]

Supplementary Materials

The complete mitochondrial genome of *Gobio huanghensis* (Cypriniformes: Cyprinidae) in the Yellow River, China

Supplementary Material legends

Supplementary Material S1. Primer sequences for PCR.
Supplementary Material S2. Results of PCR Amplification.
Supplementary Material S3. PCR sequencing results of 16 pairs of primers.
Supplementary Material S4. 16 pairs of primers sequencing assembly diagram.
Supplementary Material S5. Complete mitochondrial genome map.

Supplementary Material S1　Primer sequences for PCR.

Site name	Primer name	Primer sequence	Product size	TM value
m1-1	m1-1-F	ATCTATTACTTCTTCACCTTACCCTT	1232	60
m1-1	m1-1-R	GTTGACAGGGGAGAGTGACG	1232	60
m1-2	m1-2-F	CCCCTAGAGGAGCCTGTTC	1272	60
m1-2	m1-2-R	TGGTTGAGTTCGTTATTTTCCAG	1272	60
m1-3	m1-3-F	GCCTAAAAGCAGCCATCTAAAC	1275	60
m1-3	m1-3-R	GGAAGGCTACCGCTAAAAGG	1275	60
m1-4	m1-4-F	CAAATAAATTAGATAAAGGGAGGGC	1277	60
m1-4	m1-4-R	AAGCTTAATTAGCTGACCCCATC	1277	60
m1-5	m1-5-F	CACTGGCCCTGGTATTATGAC	1277	60
m1-5	m1-5-R	TTGTAGTGGAGAGGGTTAATGG	1277	60
m1-6	m1-6-F	TGCCTTGGTCCTACTGTCAC	1376	60
m1-6	m1-6-R	CCCTGCTGGATCAAAGAATG	1376	60
m1-7	m1-7-F	ACTTGCGGGAAATCTTGCTC	1315	60
m1-7	m1-7-R	GCCCTGGGCTTAAGACATAC	1315	60
m1-8	m1-8-F	GAATGACTCCACGGCTGC	1278	60
m1-8	m1-8-R	TTAGTCATCGGGAGGGAGG	1278	60
m1-9	m1-9-F	TCTTTCTTACCATCATCCCTACC	1278	60
m1-9	m1-9-R	TCCGAGTGACTGGATAGCTTG	1278	60
m1-10	m1-10-F	ATTACATCAGAAGTGTTCTTCTTCC	1292	60
m1-10	m1-10-R	TTTTGTAGGCGGTCTGTTCC	1292	60
m1-11	m1-11-F	AGCATTTCACCGTACTCACC	1255	60

m1-11	m1-11-R	GGCTGTGTGTTCGTTCGTAG	1255	60
m1-12	m1-12-F	CGGGTCTATTTGCCTACGAC	1275	60
m1-12	m1-12-R	CTCGGCCGTATCACCAC	1275	60
m1-13	m1-13-F	CCCTATTGCACTGTATGTCACC	1262	60
m1-13	m1-13-R	TGTTGGTTATGGCTGTTAATTCC	1262	60
m1-14	m1-14-F	CCCCATCAACGAGAACAATC	1268	60
m1-14	m1-14-R	TTGAAATGTCAGAGGTATAGTGCATAG	1268	60
m1-15	m1-15-F	AATGACTTGAAGAACCACCG	1277	60
m1-15	m1-15-R	TTAAAATCTCCCTTTTCTGGG	1277	60
m1-16	m1-16-F	CCCCCGCCAATCCAATAGTT	1366	60
m1-16	m1-16-R	AGGTGATTTATGCGATGGGCT	1366	60

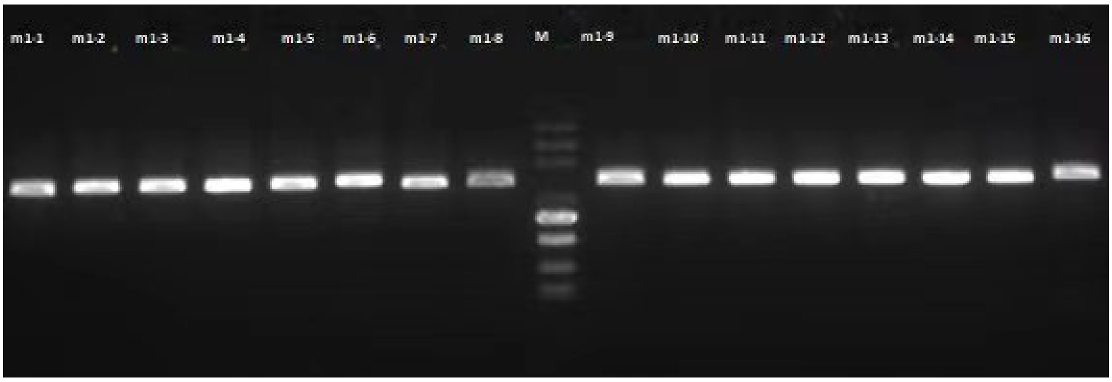

Supplementary Material S2. Results of PCR Amplification of 16 primers were designed based on reference genomes of *Rhinogobio cylindricus* and *R. ventralis*. They correspond to m1-1-m1-16 in Supplementary Material S1.

Supplementary Material S3. PCR sequencing results of 16 pairs of primers, numbered corresponding to Supplementary Material S1.

m1-1-F

ATAATCTTCCAATTGCCCCCTTTTGGTTTCTGCGCGACAAACCCCCCTACCCCCTACGCT
CAGCAAATCCTGTTATCCTTGTCAAACCCCGAAACCAAGGAAGGTTCGAGAACGTGCA
GACTAACAAGTTGGGATATGAATTAGCCATCGCATTATATATATATACATGCATGCCGCGC
TAACCCGTCGCATAAATCACCTAAATATTAGCCCAAATAACTCGATTGAAATTTTTAAAG
ATTTCTCAATGCTAAAAAATCCAACATTTTATAGTGCTAGCGTAGCTTAATACAAAGCAT
AACACTGAAGATGTTAAGATGGGCCCTAGTAAGCTCCGCATGCACAAAGGCATGGTCC
TGACCTTATTATCAGCTTTAACCCAACTTACACATGCAAGTCTCCGCAGTCCCGTGAATA
TGCCCTCAATCCCCTGCCCGGGGACGAGGAGCGGGCATCAGGCACAAACTTTAGCCCA
AGACGCCTAGCCAAGCCACACCCCCAAGGGAATTCAGCAGTGATAGACATTAGGCCAT

GAGTGAAAACTTGACTCAGTCAGGGTTAAGAGGGCCGGTAAAACTCGTGCCAGCCAC
CGCGGTTAAACGAGAGGCCCTAGTTGATATTATCACGGCGTAAAGGGTGGTTAAGGAA
TGTAAAATAATAAAGCCAAATGGCCCTTTGGCCGTCATACGCTTCTAGGTGTCCGAAGC
CCTATTATACGAAAGTAGCTTTAGTAAAATCCACCTGACCCCACGAAAGCTGAGAAAC
AAACTGGGATTAGATACCCCACTATGCTCAGCTATAAACCTAGACGTCCACATACAATT
AGACGTCCGCCGAAAAATGCTACGCATAACCTAGACGTCCACATACAATTAGACGTCC
GCCCGGGTACTACGAGCATCAGCTTGAAACCCAAAGGACCTGACGGTGCCTTAGACCC
CCCTAGAGGAGCCTGTTCTAGAACCGATAACCCCCGTTAAACCTCACCACTTCTAGCCA
CCCCAGCCTATATACCGCCGTCGCCAGCTTACCCTGTGAAGGCAATAAAAGTAAGCAA
AATGGGCACAACCCAGAACGTCAGGTCGAGGTGTAGCGTACGAAGTGGGAAGAAATG
GGCTACATTTTCTACTCATAGAACACTACGAATATGCAACATGAAATAGTGCTTGAAGG
AGGATTTAGTAGTAAAAAGGAAGCAGAGTGTCCTTTTGAACCCGGCTCTAAGGCGCGT
ACACACCGCCCGTCACTCTCCCCCTGTCAACA

m1-1-f

AAAAATGCTACGCATAACCTAGACGTCCACATACAATTAGACGTCCGCCCGGGTACTAC
GAGCATCAGCTTGAAACCCAAAGGACCTGACGGTGCCTTAGACCCCCCTAGAGGAGC
CTGTTCTAGAACCGATAACCCCCGTTAAACCTCACCACTTCTAGCCACCCCAGCCTATAT
ACCGCCGTCGCCAGCTTACCCTGTGAAGGCAATAAAAGTAAGCAAAATGGGCACAAC
CCAGAACGTCAGGTCGAGGTGTAGCGTACGAAGTGGGAAGAAATGGGCTACATTTTCT
ACTCATAGAACACTACGAATATGCAACATGAAATAGTGCTTGAAGGAGGATTTAGTAGT
AAAAAGGAAGCAGAGTGTCCTTTTGAACCCGGCTCTAAGGCGCGTACACACCGCCCG
TCACTCTCCCCCTGTCAACA

m1-1-R

TGGCGGCCGCGCACACCACGTGGCAATACATATTAATAGATGATTTGCATTGAAGTGTT
TTTGTCGTATATATATTAACAATGATGCTTATCGTACTCTCCCTGTCAACACAGTAGCCCA
TTTCTTCCCACTTCGTACGCTACACCTCGACCTGACGTTCTGGGTTGTGCCCATTTTGCT
TACTTTTATTGCCTTCACAGGGTAAGCTGGCGACGGCGGTATATAGGCTGGGGTGGCTA
GAAGTGGTGAGGTTTAACGGGGGTTATCGGTTCTAGAACAGGCTCCTCTAGGGGGGTC
TAAGGCACCGTCAGGTCCTTTGGGTTTCAAGCTGATGCTCGTAGTACCCGGGCGGACG
TCTAATTGTATGTGGACGTCTAGGTTTATAGCTGAGCATAGTGGGGTATCTAATCCCAGT
TTGTTTCTCAGCTTTCGTGGGGTCAGGTGGATTTTACTAAAGCTACTTTCGTATAATAGG
GCTTCGGACACCTAGAAGCGTATGACGGCCAAAGGGCCATTTGGCTTTATTATTTTACA
TTCCTTAACCACCCTTTACGCCGTGATAATATCAACTAGGGCCTCTCGTTTAACCGCGGT
GGCTGGCACGAGTTTTACCGGCCCTCTTAACCCTGACTGAGTCAAGTTTTCACTCATGG
CCTAATGTCTATCACTGCTGAATTCCCTTGGGGGTGTGGCTTGGCTAGGCGTCTTGGGC
TAAAGTTTGTGCCTGATGCCCGCTCCTCGTCCCCGGGCAGGGGATTGAGGGCATATTCA
CGGGACTGCGGAGACTTGCATGTGTAAGTTGGGTTAAAGCTGATAA

m1-2-F

TGGGCATCCGTTAGCTCACCACTTCTAGCCACCCCAGCCTATATACCGCCGTCGCCAG
CTTACCCTGTGAAGGCAATAAAAGTAAGCAAAATGGGCACAACCCAGAACGTCAGGT
CGAGGTGTAGCGTACGAAGTGGGAAGAAATGGGCTACATTTTCTACTCATAGAACAC
TACGAATATGCAACATGAAATAGTGCTTGAAGGAGGATTTAGTAGTAAAAAGGAAGC
AGAGTGTCCTTTTGAACCCGGCTCTAAGGCGCGTACACACCGCCCGTCACTCTCCCCT
GTCAATACGCAATAAAGATTCTTAACACCAAAGCACTGACAAGGGGAGGCAAGTCGT

AACATGGTAAGTGTACCGGAAGGTGCACTTGGATTAAACCCAGGGCGTGGCTGAGTT
AGTTAAGCATCTCACTTACACCGAGAAGACATCCATGCAAATTGGGTCGCCCTGAGC
CAAACAGCTAGCCTAATTACCGATATAACACAACAATGTTTATAATAAAGCACAACA
TAACACTAAAAACTAAACCATTTTTTTACCTGAGTATGGGAGACAGAAAAGGTTCAA
CCAAGCGCAATAGAAAAAGTACCGCAAGGGAAAGCTGAAAGAGAAATGAAATAAAC
CATATAAGCACCAAAAAACAAAGACTAAACCTTGTACCTTTTGCATCATGATTTAGCC
AGAACCCTCAAGCAAAGAGACCTTTAGTTTGAAACCCCGAAACCAGGTGAGCTACCC
CGAGACAGCCTATATAACTTAGGGCTAACCCGTCTCTGTGGCAAAAGAGTGGGAAGA
GCTCCGGGTAGAAGTGACAGACCTACCGAACCTGGTGATAGCTGG

m1-2-R

AACACTTGAATACCAAGCGCGACTACTCCTTGTGCTTGGAGAGAACAGGTCTTCTTGT
TACTCATTTTAGCATAATTTCTCCCATGCGGGCATGGGGCAGCCTAATATTACTAGGG
GAATAAGATTTTTTATCAATATAATAAACTTTTTTCTGTCTGAGCTTTAACGCTTTCCG
TTTAGATGGCTGCTTCTAGGCCCACTAGAACAGTGTGTTTTAAGTATTATGATCTTTAT
CCTCCTTAAATAAGGTTGTATCCTTCGTTAGAGGGGCTGTACCCCCTCTAACTAACTC
CCGTGTATCTCCCTTGAAAACTACCTTAATAAAACGTGTTTTGGTTTGGGGGGCACGG
GGCTGAACTTCTATCCATTTCTTAGGCAACCAGCTATCACCAGGTTCGGTAGGTCTGT
CACTTCTACCCGGAGCTCTTCCCACTCTTTTGCCACAGAGACGGGTTAGCCCTAAGTT
ATATAGGCTGTCTCGGGGTAGCTCACCTGGTTTCGGGGTTTCAAACTAAAGGTCTCTT
TGCTTGAGGGTTCTGGCTAAATCATGATGCAAAAGGTACAAGGTTTAGTCTTTGTTTT
TTGGTGCTTATATGGTTTATTTCATTTCTCTTTCAGCTTTCCCTTGCGGTACTTTTTCTA
TTGCGCTTGGTTGAACCTTTTCTGTCTCCCATACTCAGGTAAAAAAATGGTTTAGTTTT
TAGTGTTATGTTGTGCTTTATTATAAACATTGTTGTGTTATATCGGTAATTAGGCTAGC
TGTTTGGCTCAGGGCGACCCAATTTGCATGGATGTCTTCTCGGTGTAAGTGAGATGCT
TAACTAACTC

m1-3-F

CAAGACTAGCTAGACAGACTAATGTTTATTATATTGATAAAAAATCTTATTCCCCTAG
TAATATTAGGCTGCCCCATGCCCGCATGGGAGAAATTATGCTAAAATGAGTAACAAG
AAGACCTGTTCTTCTCCAAGCACAAGTGTAAGCCAGATCGGACAGACCACTGGAAAA
TAACGAACCCAACCAAAGAGAGTATTGCGGACAATAAAGAGACCAAGAAAACCCCG
CAGCTAATTAATCGTTAACCCCACACTGGAGTGCTATTGTAAAGGAAAGACTAAAAG
AAAGGGAAGGAACTCGGCAAACACAAGCCTCGCCTGTTTACCAAAAACATCGCCTCC
TGCAATAAATAAGTATAGGAGGTCCAGCCTGCCCAGTGACTACGGGTTCAACGGCCG
CGGTATTTTGACCGTGCAAAGGTAGCGCAATCACTTGTCTTTTAAATAGAGACCTGTA
TGAATGGCCAAACGAGGGCTTAACTGTCTCCCCCTTCAAGTCAGTGAAATTGATCTAT
CCGTGCAGAAGCGGGTATAAAAATACAAGACGAGAAGACCCTTTGGAGCTTAAGGTA
CAAAATTCAGCCACGTCAAACAACCCCGCAAAAAGTGAAAACTTAGTGGAACGTGAA
ATTTTACCTTCGGTTGGGGCGACCGCGGAGGAAAATACAGCCTCCGAGTGGAATGGG
GCAAAACCCCTAAAACCATGAGAAACATCTCTAAGCCGCAGAACATCTGACCAAAAA
TGATCCGGCCAACCAGCCGATCAACGAACCAAGTTACCCTAGGGATAACAGCGCAAT
CCTCTCCCAGAGTCCATATCGACGAGGGGGTTTACGACCTCGATGTTG

m1-3-R

GTTAACCTGTCCGCAGAGGGTTATTAAGTGGTTTATTAAGGTGTTCAGCATAAACTGG
GAAGAGGATTTGAACCTCTGGTTTAAAGGGCTTAGGCCTTTCGCAATTTACCATGCTC

TGCCACCCCAGTATGCCCTTATCTTGGGCGGCAGGGGTTTCGGCCCTCCCTTTATTTAA
TTTATTTGTTTTCATCAATTAGGGGTGGGGCGTGCTTCAAGTATGGGCCCCTCTTTTCC
GATCCTTTCGTACTAGGAAAAGTAGCGTTACAGATAGAAACTGACCTGGATTACTCCG
GTCTGAACTCAGATCACGTAGGACTTTAATCGTTGAACAAACGAACCCTTAATAGCG
GCTGCACCATTAGGATGTCCTGATCCAACATCGAGGTCGTAAACCCCCTCGTCGATAT
GGACTCTGGGAGAGGATTGCGCTGTTATCCCTAGGGTAACTTGGTTCGTTGATCGGCT
GGTTGGCCGGATCATTTTTGGTCAGATGTTCTGCGGCTTAGAGATGTTTCTCATGGTTT
TAGGGGTTTTGCCCCATTCCACTCGGAGGCTGTATTTTCCTCCGCGGTCGCCCCAACC
GAAGGTAAAATTTCACGTTCCACTAAGTTTTCACTTTTTGCGGGGTTGTTTGACGTGG
CTGAATTTTGTACCTTAAGCTCCAAAGGGTCTTCTCGTCTTGTATTTTTATACCCGCTT
CTGCACGGATAGATCAATTTCACTGACTTGAAGGGGGAGACAGTTAAGCCCTCGTTTG
GCCATTCATACAGGTCTCTATTTAAAAGACAAGTGATTGCGCTACCTTTGCACGGTCA
AAATACCG
m1-4-F
TCGTGGTGTCTTCTGCATTATGTTGCAGGAGAGGGCATACTGGGGTGGCAGAGCATG
GTAAATTGCGAAAGGCCTAAGCCCTTTAAACCAGAGGTTCAAATCCTCTTCCCAGTTT
ATGCTGAACACCTTAATAAACCACTTAATTAACCCTCTAGCCTACATTGTCCCTGTCCT
TTTAGCAGTAGCCTTTCTTACGCTACTTGAGCGAAAAGTCCTAGGATATATGCAACTA
CGAAAAGGGCCTAACGTCGTAGGGCCCTACGGGCTATTACAACCAATTGCTGACGGG
GTAAAACTATTTATTAAGGAACCCGTCCGCCCCTCCACATCCTCCCCATTTCTGTTTTT
AGCGACCCCTATTCTTGCATTGACCCTAGCCATGACGCTATGAGCACCTATACCTATA
CCCTACCCAGTAATTGACCTCAACCTAGGAGTTCTGTTTATCCTAGCCCTCTCAAGCCT
CGCAGTATACTCCATCTTAGGATCAGGGTGAGCATCAAATTCAAAGTATGCGCTAATT
GGAGCCCTACGAGCAGTAGCCCAAACAATTTCGTATGAGGTAAGCCTTGGGCTAATC
CTGCTCTCCGTAATTATTTTTTCCGGGGGGTACACCCTGCAGACATTTAATACAACTC
AAGAGAGCATTTGACTTTTAGCCCCAGCATGACCCCTAGCTGCAATATGATATATTTC
AACACTAGCTGAAACAAACCGGGCACCTTTCGACCTAACGGAGGGGGAATCAGAACT
AGTTTCTGGCTTCAACGTAGAATATGCAGGGGGCCCATTTGCCCTTTTTTTCCTCGCAG
AATATGC
m1-4-R
CCTAACCGTATCTAAGTGAGAGGAGCACTAAGAGTTTTGATCTCTTGGGCATGGGTTC
GACCCCCTTCTTTCTAAGAACTGAGGGGATTTTAACCCCTATCAGCCACTCTATCAAA
GTGGTCCCTGAGCATTCGGGCACAGTTCCTGAACTACAGCTGTGGAGGAAGGCCCGC
TAGTGCAACTGGTAGAGCAATATGTCATAGTACTAGGGCCAGTGTTAGTGGGAGGAA
GTTTTTCCATACAAGATGCATAAGTTGATCATACCGGAATCGTGGGTAAGATGCCCGG
ACTCAAAGAAAAACTACTGATAAAAACGCAGCTTTAACTATCAGGCTAATTGTTGTTA
GTTCAGGTATGTAGGGGAAGTGGGATGCCCCTAAGAACAGGACGGCTGATAAGGTAT
TTATTAGTAAAATGTTAGCATATTCTGCGAGGAAAAAAAGGGCAAATGGGCCCCCTG
CATATTCTACGTTGAAGCCAGAAACTAGTTCTGATTCCCCCTCCGTTAGGTCGAAAGG
TGCCCGGTTTGTTTCAGCTAGTGTTGAAATATATCATATTGCAGCTAGGGGTCATGCT
GGGGCTAAAAGTCAAATGCTCTCTTGAGTTGTATTAAATGTCTGCAGGGTGTACCCCC
CGGAAAAAATAATTACGGAGAGCAGGATTAGCCCAAGGCTTACCTCATACGAAATTG
TTTGGGCTACTGCTCGTAGGGCTCCAATTAGCGCATACTTTGAATTTGATGCTCACCCT
GATCCTAAGATGGAGTATACTGCGAGGCTTGAGAGGGCTAGGATAAACAGAACTCCT

AGGTTGAGGTCAAT
m1-5-F
GCAGTACGCTGACTAGCGGGCTTCCTCCACAGCTGTAGTTCAGGAACTGTGCCCGAAT
GCTCAGGGACCACTTTGATAGAGTGGCTGATAGGGGTTAAAATCCCCTCAGTTCTTAG
AAAGAAGGGGGTCGAACCCATGCCCAAGAGATCAAAACTCTTAGTGCTTCCTCTACA
CCACTTTCTAAGATGGGGTCAGCTAATTAAGCTTTCGGGCCCATACCCCGAACATGAC
GGTTAAAGTCCCTCCTCCATCAATGAACCCCTACGTACTAATAATTCTACTATCCAGC
CTAGGATTAGGGACCACCCTGACCTTTGCCAGCTCTCACTGACTGCTAGCTTGAATAG
GGTTAGAAATTAATACCCTAGCAATCGTGCCCTTAATAGCGCAGCATCACCACCCCCG
AGCAGTAGAGGCCACTACAAAGTACTTCCTTACCCAGGCGACCGCAGCGGCAATGAT
TCTATTTGCAAGCACGACAAACGCCTGAATTACCGGCGAATGAGATATAAACAATAT
ATCAAACCCAATTGCCAGTGCAATAGTTATTACCGCCCTTGCACTTAAAATTGGACTA
GCGCCAATACACTTCTGGATACCAGAAGTTCTACAAGGGCTAGACCTACTCACCGGTC
TAATTTTATCAACTTGACAAAAACTAGCCCCCCTCGCCCTTATTATCCAAACAGCCCA
AGCCATCGACCCACTTCTGCTAACCTCACTAGGACTTATATCTACATTAGCCGGAGGA
TGGGGCGGGCTGAACCAAACTCAACTCCGGAAAATCCTAGCCTACTCCTCTATTGCA
m1-5-R
ACCGGCTTGGGTGCTTGACTCGTCAGGGGGTATTGAATTGGCCGTATTGGGGGACAC
GGTAAGCGTTATTGCGTAGCAGAGTCGGAGATAAAAATATAGGCTAAGCAAGGCAGC
TAGGGCTATAACTGTAGCAGTAAGAGGAAAATTCTGTTTTGCCAGTTCCTGAAGGATC
AATCACTTTGGTATGAATCCTGTTAGTGGGGGGAGTCCCCCTAGTGACAGTAAAACTA
AGGCAGTTGTTGCTGTTAAGACGGGGCTGTTTGATCAAGTCATTGCTAGGGTGTTAAT
TTTTGTGGCGGAGGATGATTTTAGGGTAAGAAATGCTGCTGAAGTTATGAAGATGTA
AGTTCCTAGCGCGAGGAGTGTGAGTTGGGGGGCATATTGAAGAACAATAACTATTCA
GCCCATGTGTGCAATAGAGGAGTAGGCTAGGATTTTCCGGAGTTGAGTTTGGTTCAGC
CCGCCCCATCCTCCGGCTAATGTAGATATAAGTCCTAGTGAGGTTAGCAGAAGTGGGT
CGATGGCTTGGGCTGTTTGGATAATAAGGGCGAGGGGGGCTAGTTTTTGTCAAGTTGA
TAAAATTAGACCGGTGAGTAGGTCTAGCCCTTGTAGAACTTCTGGTATCCAGAAGTGT
ATTGGCGCTAGTCCAATTTTAAGTGCAAGGGCGGTAATAACTATTGCACTGGCAATTG
GGTTTGATATATTGTTTATATCTCATTCGCCGGTAATTCAGGCGTTTGTCGTGCTTGCA
AATAGAATCATTGCCGCTGCGGTCGCCTGGGTAAGGAAGTACTTTGTAGTGGCCTCTA
CTGC
m1-6-F
TCATCATACTACCACTAAAGGATTCATACCAAAGTGATTGATCCTTCAGGAACTGGCA
AAACAGAATTTTCCTCTTACTGCTACAGTTATAGCCCTAGCTGCCTTGCTTAGCCTATA
TTTTTATCTCCGACTCTGCTACGCAATAACGCTTACCGTGTCCCCCAATACGGCCAATT
CAATTACCCCCTGACGAGTTCAAGCAACCCAAAGCTCCCTCCCATTAACCCTGTCCAC
CACAATTGCGCTAGGTCTTTTACCCGTAACCCCAGCTATTCTAATGCTGGCCACCTAG
GGACTTAGGATAACATTAGACCAAGAGCCTTCAAAGCTCTAAGCAGAAGTGAAAATC
TTCTAGTCCCTGGATAAGACCTACAAGAGTCTATCTTGCATTTTCTGATTGCAAGTCA
AATGTTTTTATTAAACTAAGGCCTTACTAGATGGGAAGGCCTCGATCCTACAAACTCT
TAGTTAACAGCTAAGCGCTCAAGCCAGCGAGCATCCATCTACTTTCCCGCCGTTAGCC
TAGTAAGGCGGGAAAGCCCCGGCAGGGTATTAATCTACGTCTTTGGATTTGCAATCCA
ACATGCTTCTTCACCACGGGGCTGATGATAAGGAGAGGACTTAAACCTCTGTCTTCGG

GGCTACAACCCACCGCCTAAATACTCGGCTACCCTACCTGTGGCAATTACGCGCTGAT
TCTTTTCTACAAACCACAAAGACATTGGTACCCTTTATCTTGTATTTGGTGCCTGAGCC
GGAATAGTGGGGACTGCTTTAAGCCTCCTCATTCGAGCTGAGCTGAGTCAACCTGGCT
CACTTCTAGGTGATGATCAAATTTAA

m1-6-R

TTAATATAAGCATTCGATCCGTAGAGCATCGTAATTCCGGCAGCCAGAACCGGGAGT
GAAAGAAGTAGGAGTACCGCTGTTACAAGTACAGCCCATACAAATAGGGGAGTTTGA
TATTGGGAAATGGCTGGGGGCTTCATGTTAATAGTGGTGGTAATAAAATTAATGGCCC
CTAAAATTGATGAAACACCCGCTAGGTGAAGCGAAAAAATAGTGAGGTCTACTGAGG
CTCCTGCATGAGCAAGATTTCCCGCAAGTGGGGGATAAACTGTTCACCCTGTCCCAGC
CCCGGCCTCGACTCCGGAAGAGGCTAGTAGCAGAAGGAATGATGGGGGGAGAAGTC
AGAAGCTTATGTTGTTTATTCGGGGGAATGCCATGTCTGGGGCCCCGATTATTAGCGG
TACAAGCCAGTTTCCAAACCCTCCAATAAGAATTGGTATTACTATAAAGAAAATTATT
ACGAAGGCGTGTGCAGTGACGATTACATTATAAATTTGATCATCACCTAGAAGTGAG
CCAGGTTGACTCAGCTCAGCTCGAATGAGGAGGCTTAAAGCAGTCCCCACTATTCCG
GCTCAGGCACCAAATACAAGATAAAGGGTACCAATGTCTTTGTGGTTTGTAGAAAAG
AATCAGCGCGTAATTGCCACAGGTAGGGTAGCCGAGTATTTAGGCGGTGGGTTGTAG
CCCCGAAGACAGAGGTTTAAGTCCTCTCCTTATCATCAGCCCCGTGGTGAAGAAGCAT
GTTGGATTGCAAATCCAAAGACGTAGATTAATACCCTGCCGGGGCTTTCCCGCCTTAC
TAGGCTAACGGCGAGAAAGTAGATGGATGCTCGCTGGCTTGAGCGCTTAG

m1-7-F

ATGATCAGTAGACTCCTATTTTTTCGCTTCACCTAGCGGGTGTTTCATCAATTTTAGGG
GCCATTAATTTTATTACCACCACTATTAACATGAAGCCCCCAGCCATTTCCCAATATC
AAACTCCCCTATTTGTATGGGCTGTACTTGTAACAGCGGTACTCCTACTTCTTTCACTC
CCGGTTCTGGCTGCCGGAATTACGATGCTTCTTACGGATCGAAATCTTAACACTACAT
TCTTTGATCCAGCAGGGGGAGGAGACCCAATCCTTTACCAACACCTATTCTGATTCTT
CGGTCACCCAGAAGTATACATCCTTATTTTACCAGGATTTGGCATCATTTCACATGTT
GTAGCTTACTACTCAGGCAAAAAAGAGCCATTTGGGTATATGGGAATAGTATGAGCT
ATGATGGCTATTGGTCTCCTAGGCTTTATTGTGTGAGCACACCATATGTTCACCGTAG
GAATAGACGTAGACACCCGTGCCTACTTCACATCCGCAACAATAATTATTGCTATTCC
AACAGGGGTAAAAGTATTTAGCTGACTTGCTACACTACATGGAGGCTCTATTAAATG
AGACACGCCTATGCTATGAGCCCTCGGGTTCATCTTCCTTTTTACTGTGGGAGGACTA
ACAGGAATTGTCCTAGCCAATTCTTCACTAGATATTGTACTCCACGACACATACTACG
TAGTTGCACACTTCCACTATGTATTATCAATAGGGGCCGTATTTGCTATCATAGCAGC
CTTTGTCCACTGATTCCCGCTATTTTCAGGATA

m1-7-R

ACGACATCTGTATTTACCTTGACAGGTGATGTAATTTGCATTTTACTAATGTCTTCAGA
AGAAAGTGACAGAGTGGTTATGTGACTGGCTTGAAACCAGTACATGGGGGTTCAATT
CCTCCCTTTCTCGTTAGTTTGATTGAACTCGTACGAACGCTGGTTCCTCAAATGTGTGG
TAAGGGGGAGGGCAGCCGTGGAGTCATTCTACGTTCGTCATGGTTAGTTCTACTGAGG
ATACCTCCCGTTTGGCGGCGAAGGCCTCTCATAGAATAAATAGGAATATAATTACTGC
GACCAGGGAGATAAGTGATCCGATAGATGACACTGTATTTCACAGGGCGTAGGCGTC
TGGGTAGTCAGAGTATCGTCGTGGCATTCCTGCTAAACCTAAAAAATGTTGGGGGAA
GAATGTCAGATTTACGCCAATAAACATTACACCAAAGTGGATTTTTGTTCAAGTGTCA

TTTAGGGTGTATCCTGAAAATAGCGGGAATCAGTGGACAAAGGCTGCTATGATAGCA
AATACGGCCCCTATTGATAATACATAGTGGAAGTGTGCAACTACGTAGTATGTGTCGT
GGAGTACAATATCTAGTGAAGAATTGGCTAGGACAATTCCTGTTAGTCCTCCCACAGT
AAAAAGGAAGATGAACCCGAGGGCTCATAGCATAGGCGTGTCTCATTTAATAGAGCC
TCCATGTAGTGTAGCAAGTCAGCTAAATACTTTTACCCCTGTTGGAATAGCAATAATT
ATTGTTGCGGATGTGAAGTAGGCACGGGTGTCTACGTCTATTCCTACGGTGAACATAT
G

m1-8-F

AACATTCGTCGAGTTCATCAACTAACGAGAAAGGGAGGAATTGAACCCCCATGTACT
GGTTTCAAGCCAGTCACATAACCACTCTGTCACTTTCTTCTGAAGACATTAGTAAAAT
GCAAATTACATCACCTTGTCAAGGTGAAATTACAGGTTAAATCCCTGTATGTCTTAAG
CCTAAGGCTTAATGGCACATCCCACACAACTAGGATTCCAAGACGCGGCATCACCCG
TTATAGAAGAGCTTCTTCACTTCCACGACCATGCTCTAATAATTGTATTTTTAATTAGT
ACCCTAGTACTTTACATTATTATTGCGATGGTTTCAACTAAACTTACCAACAAATACA
TCTTAGACTCACAAGAAATCGAAATTGTATGAACCGTTTTACCAGCCGTCATTCTAGT
TTTGATTGCACTACCCTCCCTTCGAATTTTGTATCTTATAGACGAAATTAATGACCCCC
ACCTAACAATTAAAGCCATGGGACACCAGTGATACTGAAGTTACGAATACACGGACT
ATGAAGACCTCGGCTTCGATTCCTATATAATTCCGACTCAAGACCTCACACCAGGTCA
ATTCCGACTACTAGAGACAGATCACCGAATGGTTGTCCCAATAGAGTCACCCATTCGT
GTGCTAGTATCCGCTGAAGACGTTTTACACTCATGAGCCGTCCCATCTTTAGGCGTAA
AAATAGACGCAGTGCCAGGGCGACTAAACCAAACTGCCTTTATTGCCTCCCGCCCAG
GGCTCTTCTATGGACAATGCTCTGAAATCTGTGGTGCCAACCACAGCTTTATGCCAAT
TGTAGTTGAAGCCGTTCCACTAGAACACTTTGAAAGCTGATCCTCATTAATACTAGAA
GACGCCTCACTAGAAAGCTAATTATTGGAACAAAGCGTTGGCCTTTTAAGCCA

m1-8-R

GTAAGAGCATGCGATTGCGACGGCATAAGTGGAATTCCTAGGAAGGAAGGGCTTGCA
AATTGGTCAAAAAAGCTTGTTATCATGGTCAGTCTCAAGGCTCAGTTTTGTGTTTTTCT
TCGCTTATTGGCACGGGTTCGTTTGGTGCCGTATGGCTTAAAATTTGGTGGGGATGA
TGGTAAGAAATACGATTCATGAGAACACTAGAATAGCAAATCAAGGGTTGGGGTTTA
ATTGGGGCATTTCACTAGAGGTGGTCGGTAGTCACCAAACTTTGGCTTAAAAGGCCA
ACGCTTTGTCCAATAATTAGCTTTCTAGTGAGGCGTCTTCTAGTATTAATGAGGATCA
GCTTTCAAAGTGTTCTAGTGGAACGGCTTCAACTACAATTGGCATAAAGCTGTGGTTG
GCACCACAGATTTCAGAGCATTGTCCATAGAAGAGCCCTGGGCGGGAGGCAATAAAG
GCAGTTTGGTTTAGTCGCCCTGGCACTGCGTCTATTTTTACGCCTAAAGATGGGACGG
CTCATGAGTGTAAAACGTCTTCAGCGGATACTAGCACACGAATGGGTGACTCTATTGG
GACAACCATTCGGTGATCTGTCTCTAGTAGTCGGAATTGACCTGGTGTGAGGTCTTGA
GTCGGAATTATATAGGAATCGAAGCCGAGGTCTTCATAGTCCGTGTATTCGTAACTTC
AGTATCACTGGTGTCCCATGGCTTTAATTGTTAGGTGGGGGTCATTAATTTCGTCTATA
AGATACAAAATTCGAAGGGAGGGTAGTGCAATCAAAACTAGAATGACGGCTGGTAA
AACGGTTCATACAATTTCGATTTCTTGTGAGTCTAAGATGTATTTGTTGGTAAGTTTAG
TTGAAACCATCGCAATAAT

m1-9-F

GGCACCTCTCTGATAGCTACGGCACCAACGAACCCGTGCCAATAAGCGAAGAAAAAC
ACAAAACTGAGCCTTGAGACTGACCATGATAACAAGCTTTTTTGACCAATTTGCAAGC

CCTTCCTTCCTAGGAATTCCACTTATTGCCGTCGCAATCGCACTCCCCTGAATACTATT
TCCAACACCCCCATCCCGATGATTAAACAACCGACTAATTACTCTCCAAACATGATTC
ATTAACCGATTCACCAACCAACTGTTACTACCCCTAAATACAGGAGGACATAAATGA
GCTTTATTATTTGCCTCTTTAATAGTCTTTTTAATTACTATTAATATGTTAGGCCTTCTC
CCATACACTTTCACGCCAACAACTCAATTATCTTTAAACATGGGATTTGCTGTGCCCTT
ATGACTTGCTACAGTAATTATTGGCATACGTAATCAACCAACCGTTGCCCTTGGACAT
CTTCTACCAGAAGGAACGCCAATTCCCCTTATTCCAGTACTAATTATTATCGAAACAA
TTAGTTTATTTATTCGACCACTAGCACTAGGGGTACGACTCACAGCTAACTTAACTGC
GGGCCATCTACTTATTCAACTAATTGCTACTGCTGTATTTGTATTAATACCTATAATAC
CAACAGTCGCAATCTTAACGGCCGCCGTTCTTTTCCTCCTTACACTTCTAGAAGTTGCA
GTTGCAATAATTCAAGCTTATGTGTTTGTACTTCTACTAAGCCTCTACCTGCAAGAAA
ACGTCTAATGGCACACCAAGCACATGCATATCATATGGTCGACCCCAGCCCATGACC
ACT

m1-9-R

TAAGACATATGATGCTGTGGTGGGCTCATGTAACTGTTACCCCTGATGCTAACAATAC
TGCTGTATTTAGAAGGGGTACTTCAAAGGGGTCTAGTGTGGTAATTCCAGTTGGTGGT
CAACATCCTCCTAGCTCAGGTGTTGGGGCTAAGCTTGAGTGGTAGAAAGCTCAGAAG
AACCCGAGGAAGAAAAACACTTCTGATGTAATAAATAAAATTATACCATAGCGCAGG
CCTTTTTGTACTGGGGGTGTATGATGTCCCTGGAAGGTACCTTCCCGAATAATGTCAC
GTCATCACTGGATTATAGTAAGAAGAAGAAGAATTAGTCCAAGGGTTATTAATGTTG
TTGAGTGGAAGTGAAACCAGATTGCTAGGCCGGATGTTATTAGTAGAGCACCGACGG
CTCCGGTTAGTGGTCATGGGCTGGGGTCGACCATATGATATGCATGTGCTTGGTGTGC
CATTAGACGTTTTCTTGCAGGTAGAGGCTTAGTAGAAGTACAAACACATAAGCTTGA
ATTATTGCAACTGCAACTTCTAGAAGTGTAAGGAGGAAAAGAACGGCGGCCGTTAAG
ATTGCGACTGTTGGTATTATAGGTATTAATACAAATACAGCAGTAGCAATTAGTTGAA
TAAGTAGATGGCCCGCAGTTAAGTTAGCTGTGAGTCGTACCCCTAGTGCTAGTGGTCG
AATAAATAAACTAATTGTTTCGATAATAATTAGTACTGGAATAAGGGGAATTGGCGTT
CCTTCTGGTAGAAGATGTCCAAGGGCAACGGTTGGTTGATTACGTATGCCAATAATTA
CTGTAGCAAGTCATAAGGGCACAGCAAATCCCATGTTTAAAGATAATTGAGTTGTTG
GCGTGAAAGTGT

m1-10-F

ACCTTACTACCGTACGCTCTGACTTCTCCACTCAAGCTTAGCCCCAACACCTGAGCTA
GGAGGATGTTGACCACCAACTGGAATTACCACACTAGACCCCTTTGAAGTACCCCTTC
TAAATACAGCAGTATTGTTAGCATCAGGGGTAACAGTTACATGAGCCCACCACAGCA
TCATGGAGGGTGAACGAAAACAAGCTATTCAATCACTCGGACTCACAATTTTATTAG
GACTTTACTTCACTGCCCTACAAGGCATAGAATACTACGAAGCACCTTTCACGATTGC
AGATGGAGTCTATGGCTCCACATTCTTTGTGGCTACAGGATTCCACGGGCTACATGTT
ATTATTGGATCAACCTTCCTGGCCGTTTGCCTCCTCCGACAAATTCAATACCACTTCAC
ATCTGAACACCACTTCGGTTTTGAGGCCGCTGCCTGATACTGACATTTTGTTGACGTA
GTATGACTATTCCTTTACGTATCCATCTACTGATGAGGCTCATATCTTTCTAGTATTAA
ATTAGTACAAGTGACTTCCAATCATTTAGTCTTGGTTGAACCCCAGGGAAAGATAATG
AACTTAATTATGACTATTCTCCTTATTACAGTAGCCCTGTCATCAGTTTTAGCAATTGT
ATCCTTCTGGTTACCACAAATGAACCCAGACGCAGAAAAGCTCTCCCCGTACGAGTGT
GGGTTCGACCCTCTGGGATCTGCCCGACTACCATTCTCATTACGATTCTTTCTAGTTGC

CATTCTTTTCCTCTTATTTGATCTAGAAATTGCCCTCCTTCTCCCCTTGGCC
m1-10-R
AAGAGGAAAAACCTTAACACTCTGCTCTGAGTGCTAGCCCTGTGCTTGCTTCACAAGC
GGAGAAGGCCAGAAGAAGTATGGGGGCAGTGGAGAAACTAGTAGATTCAAACTGTA
GTGCTCATAGGGCTAATGCAATAAATAGGGATAGTATTATGCCTTCTAAGCATAAAA
GCGCTGATAACAGGTGGGTACGGTGAAATGCTAGTCCTATTAGGCCAAGAATAAATG
CTGAACTGAAGCTGAAATGTACTGGTGTCATAAGGGGGTCGTGGACTTAAACCACAA
TTTTCTGAGCCGAAATCAGAGGTCTTATTTGGACTAACTCCCTTATTCTGCTCATTCT
AAGCCTCCTTGGGTTCACTCATAGACTAGCCCTAGGGTTAATAAAATCAGGACTGTAG
TGGCTCAAAAGAATGTTCCTGTGGGGTTGTGAAGTTGATCCCCTCAGGGCAAGGGGA
GAAGGAGGGCAATTTCTAGATCAAATAAGAGGAAAAGAATGGCAACTAGAAAGAAT
CGTAATGAGAATGGTAGTCGGGCAGATCCCAGAGGGTCGAACCCACACTCGTACGGG
GAGAGCTTTTCTGCGTCTGGGTTCATTTGTGGTAACCAGAAGGATACAATTGCTAAAA
CTGATGACAGGGCTACTGTAATAAGGAGAATAGTCATAATTAAGTTCATTATCTTTCC
CTGGGGTTCAACCAAGACTAAATGATTGGAAGTCACTTGTACTAATTTAATACTAGAA
AGATATGAGCCTCATCAGTAGATGGATACGTAAAGGAATAGTCATACTACGTCAACA
AAATGTCAG
m1-11-F
TACGCCTCATGCTTAGAGGCATAATACTATCCCTATTTATTGCATTAGCCCTATGAGC
ACTACAGTTTGAATCTACTAGTTTCTCCACTGCCCCCATACTTCTTCTGGCCTTCTCCG
CTTGTGAAGCAAGCACAGGGCTAGCACTTCTAGTAGCCACAGCCCGGACCCACGGGA
CAGACCGCCTACAAAACCTAAACCTCTTACAATGCTAAAAGTATTAATCCCAACAATC
ATGATATTCCCAACAATCTGATTAATCTCCCCAAAATGGTTATGGACGGCCACAATTA
CCCACAGCCTTTCGATTGCCCTTATTAGCCTCACATGACTAAAGTGAACATCCGAAAC
AGGATGGGCCACCTCTAATATGTACCTGGCCACAGACCCCCTATCAACCCCCCTGCTA
GTATTGACCTGCTGGCTTCTTCCACTTATAATTTTAGCCAGCCAAAACCACATTAACC
CCGAACCTATCAGCCGACAACGCCTTTATATTACACTTCTTACCTCCCTACAAACTTTC
CTAATCTTAGCCTTCGGTGCTACAGAGATCATCATGTTCTACATTATGTTTGAAGCCA
CACTTATCCCGACCTTAATTATTATTACTCGGTGGGGCAACCAAACCGAACGACTTAA
TGCCGGAACGTACTTTTTATTTTATACACTAGCAGGCTCTCTGCCGCTCCTTGTCGCGC
TACTCCTCCTACAGCAGTCCACAGGGACCCTCTCCATATTAGTAATCCAATATTCACA
GCCACTCTTATTAGACTCCTGAGGCCATAAAATCTGGTGGGCCGCTTGTCTAATCGCA
TTTCTAGTAAAGATACCGCTCTA
m1-11-R
AGGACTAAGACTTTTATTTTATACACTAGCAGGCTCTCTGCCGCTCCTTGTCGCGCTAC
TCCTCCTACAGCAGTCCACAGGGACCCTCTCCATATTAGTAATCCAATATTCACAGCC
ACTCTTATTAGACTCCTGAGGCCATAAAATCTGGTGGGCCGCTTGTCTAATCGCATTT
CTAGTAAAGATACCGCTCTATGGGGTCCACCTATGACTTCCAAAAGCACATGTAGAG
GCCCCAGTAGCAGGGTCTATAGTACTGGCAGCCGTGCTCCTCAAACTAGGGGGGTAC
GGTATAATACGAATAATGATTATACTAGACCCGCTCTCGAAACAACTAGTCTATCCTT
TTATTATTCTAGCGCTATGGGGTATTATTATGACCGGGTCTATTTGTATACGACAAAC
AGACCTTAAATCCCTAATCGCCTATTCATCGGTTAGCCACATAGGACTTGTGGCAGGT
GGCATCCTGATTCAAACCCCATGAGGATTTTCAGGAGCAATTATCCTTATAATTGCCC
ACGGATTAGTGTCCTCAATACTTTTTGTCTAGCCAACACAGCCTACGAACGACCCCC

CCCCAGCCAA
m1-12-F
GGGGACTACTGCTATCGCCTATTCATCGGTTAGCCACATAGGACTTGTGGCAGGTGGC
ATCCTGATTCAAACCCCATGAGGATTTTCAGGAGCAATTATCCTTATAATTGCCCACG
GATTAGTGTCCTCAATACTTTTTGTCTAGCCAACACAGCCTACGAACGGACACACAG
TCGAACCATAGTACTTGCCCGAGGACTACAGATAATTTCCCGCTAACAGCAGTTTGA
TGATTCATTGCCAATCTGGCTAACCTGGCCCTCCCCCCACTACCTAACTTAATAGGGG
AACTCATGATTATTACAACCCTATTCAACTGATCCCCATGAACTATTGCACTTACAGG
AGCGGGCACATTAATTACAGCTGGTTACTCCCTCTACTTATTCCTAATAACTCAACGA
GGCCCAGCACCAAACCACATCATAAATCTCCCACCATTCCACACCCGGGAGCACTTAT
TAATAGCCCTTCACCTAATCCCCGTAATTCTCCTTGTAGCAAAACCGGAACTTATGTG
GGGATGATGTTATTAGTAAGTATAGTTTAACTAAGATATTAGATTGTGATTCTAAAGA
CAGGGGTTAAAGTCCCCTTACTCACCAAGGAAGGACAGAAACCAGTAAGTACTGCTA
ATCCTTATGCACCGCGGTTAAACTCCGCGGCTTCCTCACGCTTCTGAAGGATAACAGC
TCATCCATTGGTCTTAGGAACCAAAAACTCTTGGTGCAAATCCAAGTGGAAGCTATGA
ATTCAATAACACTAATTATATCCTCCTCACTTATTTAGTTATTACAATCCTTATCATC
CCGCT
m1-12-R
AACCTTCTATTTCCACCCCCTCTCACCCAATAAATAGTTGAAACATATTATTAGCGGT
AACAAGGATAATTATAGCTACCAAAAATAGTAGGAGGTATTTAAAAAACCGGTTTAT
ATTAGGGTCGGAGTGTATATATCATAGTGCAAACTCTAAAATCGATCAGGTGACGTA
CAGGGCAATAGGGGTAAAAATAAGGGAGTAGTGGTCAAATTTAAAGCTAATATTAGT
GTCAAATATGTGTGTGTTTATTCAGTGTCAGTTTGTAGTAATGCTCTCCACCCCCTGAT
CTAAAAAGATCATAAGCGGAAGAAGGCTAATAAAGAATGCGGTGCTGACAGCATTCT
TAACACGTGTGTTTGCTCAGTTAGGGTCCTGTGGCTTTGGGTTTAATGTTATTAGTAGC
GGGATGATAAGGATTGTAATAACTAAAATAAGTGAGGAGGATATAATTAGTGTTATT
GAATTCATAGCTTCCACTTGGATTTGCACCAAGAGTTTTTGGTTCCTAAGACCAATGG
ATGAGCTGTTATCCTTCAGAAGCGTGAGGAAGCCGCGGAGTTTAACCGCGGTGCATA
AGGATTAGCAGTACTTACTGGTTTCTGTCCTTCCTTGGTGAGTAAGGGGACTTTAACC
CCTGTCTTTAGAATCACAATCTAATATCTTAGTTAAACTATACTTACTAATAACATCAT
CCCCACATAAGTTCCGGTTTTGCTACAAGGAGAATTACGGGGATTAGGTGAAGGGCT
ATTAATAAGTGCTCCCGGGTGTGGAATGGTGGGAGATTTATGATGTGGTTTGGTGCTG
GGCCTCGTTGAGTTATTAGGAATAAGTAGAGGGAGTAACCAGCTGTAATTAATGTGC
CCGCTCC
m1-13-F
GAGGACTCGTAGTTGACTATGATATATACACTCCGACCCTAATATAAACCGGTTTTTT
AAATACCTCCTACTATTTTTGGTAGCTATAATTATCCTTGTTACCGCTAATAATATGTT
TCAACTATTTATTGGGTGAGAGGGGGTTGGAATTATGTCCTTTTTACTGATTGGATGG
TGGTACGGCCGAGCGGACGCTAATACAGCAGCCCTTCAGGCTGTGATCTACAACCGG
GTGGGAGATATTGGATTAATCCTCAGCATGGCCTGATTCGCAATAAATTTAAACTCCT
GAGAAATTCAACAAATCTTCTTTCTATCAAAAAATTTTGACATGACGATCCCCTTAAT
CGGATTAATTCTAGCAGCAACAGGAAAATCGGCCCAATTTGGCCTTCATCCATGGCTA
CCTTCTGCCATGGAGGGCCCTACGCCAGTATCTGCCCTACTCCATTCCAGCACAATAG
TGGTTGCCGGGATTTTTCTATTAATTCGCCTTCACCCCCTTATAGAGAACAACAAGAC

TGCGTTGACAATTTGTCTTTGCCTAGGCGCACTAACCACGCTATTTACAGCTACCTGC
GCCCTAACCCAAAATGATATCAAAAAAATTGTAGCTTTCTCAACATCAAGTCAGCTAG
GCCTAATAATAGTTACAATTGGGCTTAATCAACCACAACTGGCATTTCTTCACATCTG
CACACACGCCTTTTTCAAGGCCATGTTATTCTTATGTTCCGGCTCAATTATTCATAGCC
TGAATGATGAACAAGATATCCGAAAAATAGGAGGTCTCCACAACTTGATACCCGCTA
CTTCAACCTACCTTACAATTGGCA

m1-13-R

GACATGTCTGATTCACTGGGTCTCAGGGTACCATGAGGGCTGCTATTTTTAGGGCCAA
GGGTATAGTCATAATGGGTGTTTTTGAGGGTGGGAAGTTGGATGTAATAATAAGTCCC
GCAACAATACTTCCTCAAGCAAGTCGTTTAATAGGGTTAATTACTAAGGGGCTGTTTT
CATTAATTGGGGATAGTGGGAGGAACCGTGGGGATCCCATAGTCACAAAGAATACAA
CTCGGAAGCTGTATACTGCGGTAAATGATGTGGCAATTAGTGTAAGGGTTAGGGCTC
AGGCGTTAAGGTGTGAGGTGTTCAGGGCTTCAATAATGGCGTCTTTTGAAAAGAACC
CGGCCAGGAAGGGAGTTCCTGTTAGTGCTAGACTGCCAATTGTAAGGTAGGTTGAAG
TAGCGGGTATCAAGTTGTGGAGACCTCCTATTTTTCGGATATCTTGTTCATCATTCAGG
CTATGAATAATTGAGCCGGAACATAAGAATAACATGGCCTTGAAAAAGGCGTGTGTG
CAGATGTGAAGAAATGCCAGTTGTGGTTGATTAAGCCCAATTGTAACTATTATTAGGC
CTAGCTGACTTGATGTTGAGAAAGCTACAATTTTTTTGATATCATTTTGGGTTAGGGC
GCAGGTAGCTGTAAATAGCGTGGTTAGTGCGCCTAGGCAAAGACAAATTGTCAACGC
AGTCTTGTTGTTCTCTATAAGGGGGTGAAGGCGAATTAATAGAAAAATCCCGGCAAC
CACTATTGTGCTGGAATGGAGTAGGGCAGATACTGGCGTAGGGCCCTCCATGGCAGA
AGGTAGC

m1-14-F

GTCGTACTTCTTTAGTATTACCCTATTAACGACTTGCTTGAGGAAGTATTGTTGCGGG
ACTTATTATTACATCCAACTTCCCACCCTCAAAAACACCCATTATGACTATACCCTTG
GCCCTAAAAATAGCAGCCCTCATGGTTACCCTTGTAGGACTCCTAGTGGCCGTGGAAT
TAACAGCCATAACCAACAAGCAAGTTAAAATTACCCCCACAATCCGCCTCCATAATTT
CTCAAACATACTAGGGTATTTTCCATCATTAATTCATCGACTACCCCCGAAACTAAAC
CTGACCTTAGGGCAGTCAATCGCCACTAAGCTCGACCAAACATGATTCGAGATCTCCG
GGCCAAAAGGGCTAGCACTAACACAAATGATAATGTCAAAAGTTACGAGTGACATCC
AACGAGGAATAATTAAAACATACCTAACCATCTTCCTGTTAACCTTGACCCTGGCCAT
CCTTTTAACAATTATTTAAACAGCCCGAAGAGTACCGCGACTTAGCCCCCGGGTTAGC
TCTAACACTACAAGAAGTGTTAGAAGCAACACTCAAGCACAAAGAACCAGCATGGCC
CCGCCAAAAGAATATATAACAGCTACACCACTAACATCACCCCGCAGCATTGAGAAC
TCTTTCAACCCATCAATAATTACCCAAGAACCTTCATACCAACCACCCCAAAACATAC
CACCTGCTAAAATCACCCCCAGTAAATAAACCAAAACGTACCCCGCAACGGAACGAC
TTCCCCAAGCTTCGGGAAAAGGCTCAGCAGCCAAAGCCGCTGAATAGGCAAACACCA
CTAGTATACCCCCTAAATAGATTAAAAAGAGAACCAAAGATAAAAAAGACCCCCCGC
ACCCCACTAAGACCCCACACCCAACTCCCGCT

m1-14-R

ATACGTACTCTGAGGTGTAGTGTATGGCCAGAAATAATCCTGTTAAGATTTGGGTAAT
TAGACAGAGCCCCAGTAGGGAGCCAAAGTTTCATCATACTGAAATATTAGATGGTGT
TGGTAGGTCAACTAGTGCGTCATTAGCGATCTTTATTAGCGGGTGGGTTTTTCGTAGG
CTTGCCATTAGTTCTTGTAGTTGAACTACAACGGTGGTTCTTCAAGTCACTGGTCTCGG

TTAAAGTCCGAGCAAGAATTATGACCTATTTTATGTTTATGTTATTGGTGGCTTTAATT
GTGGGATTAATTGCTGTTGCTTCAAATCCCACCCCTTATTTTGCTGCTCTAGGGTTAGT
GGTAGCAGCGGGAGTTGGGTGTGGGGTCTTAGTGGGGTGCGGGGGGTCTTTTTATCT
TTGGTTCTCTTTTTAATCTATTTAGGGGGTATACTAGTGGTGTTTGCCTATTCAGCGGC
TTTGGCTGCTGAGCCTTTTCCCGAAGCTTGGGGAAGTCGTTCCGTTGCGGGGTACGTT
TTGGTTTATTTACTGGGGGTGATTTTAGCAGGTGGTATGTTTTGGGGTGGTTGGTATG
AAGGTTCTTGGGTAATTATTGATGGGTTGAAAGAGTTCTCAATGCTGCGGGGTGATGT
TAGTGGTGTAGCTGTTATATATTCTTTTGGCGGGGCCATGCTGGTTCTTTGTGCTTGAG
TGTTGCTTCTAACACTTCTTGTAGTGTTAGAGCTAACCCGGGGGCTAAGTCGCGGTAC
TCTTCGGGCTGTTTAAATAATTGTTAAAAGGATGGCCAGGGTCAAGGTTAACAGGAA
GATGGTTAGGTATGTTTTAATTATTCCTCGTTGGATGTCACTCGTAACTTTTGACATTA
TCATTTGTGTTAGTGCTAGCCCTTTTGGCCCGGAGA
m1-15-F
GCAAGAATCCTGATCTAGTCGGCAAGCCTACGAAAAACCCACCCGCTAATAAAGATC
GCTAATGACGCACTAGTTGACCTACCAACACCATCTAATATTTCAGTATGATGAAACT
TTGGCTCCCTACTGGGGCTCTGTCTAATTACCCAAATCTTAACAGGATTATTTCTGGCC
ATACACTACACCTCAGACATTTCAACTGCATTCTCATCGGTCGCCCATATCTGCCGAG
ACGTAAACTACGGCTGATTTATCCGTAACGTCCACGCCAACGGAGCATCATTCTTTTT
CATCTGCATTTACATACACGTTGCCCGAGGCCTCTATTACGGATCCTACCTCTACAAA
GAAACCTGAAACATCGGAGTAGTTCTACTCCTCTTAGTTATAATAACGGCCTTCGTTG
GCTATGTTCTCCCATGAGGCCAAATGTCATTCTGAGGGGCCACAGTGATTACCAACCT
TTTATCAGCAGTCCCTTATATAGGGGATACCCTTGTCCAATGAATTTGAGGCGGTTTTT
CAGTAGATAACGCAACACTGACGCGATTCTTCGCCTTTCACTTTCTCCTGCCATTTATT
ATTGCCGCCGCAACCGTCCTCCACCTACTATTTTTACACGAAACAGGATCGAATAACC
CAGCCGGACTAAACTCTGATGCAGACAAAATCTCCTTCCACCCATACTTCTCTTATAA
GGACCTTCTTGGTTTTGTTTTGATGCTTCTAGCTCTTACATCATTAACATTATTTTCTCC
CAACTTATTGGGAGACCCGGACAACTTCACCCCCGCGAACCCGATAGTTACCCCTCCC
CACATTAAACCCGAATGATATTTCCTGTTTGCCTACGCC
m1-15-R
GGGAGATAGCTCCGATCTTCGGATTACAAGACCGATGCTTTTAGGCTAAGCTACTAGG
GCAAGCTCATTTTAATGCTTTATTTTCCAGTCAGCCTGCCACTGGGACAAGGACAAGG
AAAAGTGCAAAATACAGGACTGAAGCGACTTGACCGATGACAACATATGGATGTTCT
ACGGGCATCCCTCCAATTCATGTTAGAATAAGTATATCTGCTACGAGAGTTCAGAATA
AGAATTGTGTTAGGGGGCGGAATGTCAGGCCTCGCTGTTTTGAAGTATGTAAAACTG
GAACTACTATGAGAATTAGGATCGAGAATAATAGTGCAAGGACACCTCCAAGTTTGT
TAGGGATGGATCGTAGGATGGCGTAGGCAAACAGGAAATATCATTCGGGTTTAATGT
GGGGAGGGGTAACTATCGGGTTCGCGGGGGTGAAGTTGTCCGGGTCTCCCAATAAGT
TGGGAGAAAATAATGTTAATGATGTAAGAGCTAGAAGCATCAAAACAAAACCAAGA
AGGTCCTTATAAGAGAAGTATGGGTGGAAGGAGATTTTGTCTGCATCAGAGTTTAGTC
CGGCTGGGTTATTCGATCCTGTTTCGTGTAAAAATAGTAGGTGGAGGACGGTTGCGGC
GGCAATAATAAATGGCAGGAGAAAGTGAAAGGCGAAGAATCGCGTCAGTGTTGCGTT
ATCTACTGAAAAACCGCCTCAAATTCATTGGACAAGGGTATCCCCTATATAAGGGACT
GCTGATAAAAGGTTGGTAATCACTGTGGCCCCTCAGAATGACATTTGGCCTCATGGGA
GAACATAGCCAACGAAGGCCGTTATTATAACTAAGAGGAGTAGAACTACTCCGATGT

TTCAGGTTTCTTTGTAGAGGTAGGATCCGTAATAGAGGCCTCGGGCAACGTGTATGTA
AATGCAGATGAAAAAGAATGATGCTCCGTTGGCGTGGACGTACGGATAAATCAGCCG
TAGTTACGTCTCGGCAGATATGGGCGACCGAT

m1-16-F

GGAAACGGTTGCCTGTATTACCGATGATATTTCCTGTTTGCCTACGCCATCCTACGAT
CCATCCCTAACAAACTTGGAGGTGTCCTTGCACTATTATTCTCGATCCTAATTCTCATA
GTAGTTCCAGTTTTACATACTTCAAAACAGCGAGGCCTGACATTCCGCCCCCTAACAC
AATTCTTATTCTGAACTCTCGTAGCAGATATACTTATTCTAACATGAATTGGAGGGAT
GCCCGTAGAACATCCATATGTTGTCATCGGTCAAGTCGCTTCAGTCCTGTATTTTGCA
CTTTTCCTTGTCCTTGTCCCAGTGGCAGGCTGACTGGAAAATAAAGCATTAAAATGAG
CTTGCCCTAGTAGCTTAGCCTAAAAGCATCGGTCTTGTAATCCGAAGATCGGAGGTTA
AATTCCTCCCTAGCGCCCAGAAAAGGGAGATTTTAACTCCCACCCCTGGCTCCCAAAG
CCAGAATTCTAAATTAAACTATCTTCTGATAGTAACATGTATGGTGTAGTGCATAATA
TGCATAATATTACATAATGCAATAGTACCCATATATGTATTATCACCATTAAATTATTT
TAACCCCAAAGCAAGTACTATCGTCCTAAACGTACATAAGCTAAATATTAAAACTCA
GAAATAATTTATATTAACCTGGGAAATAGATTAATCCCTTAAAAATGGCACTCACATT
TTTCCTCGAAATACTCAACTAAGGTTTATTTTAAAAATATTAATGTAGTAAGAGACCA
CCAACTGGTTGAAATAAAGGCATACTATTAATGATAGAACCAGGGACA

m1-16-R

CTACCTACGAATATTTATATTAACCTGGGAAATAGATTAATCCCTTAAAAATGGCACTCA
CATTTTTCCTCGAAATACTCAACTAAGGTTTATTTTAAAAATATTAATGTAGTAAGAGAC
CACCAACTGGTTGAAATAAAGGCATACTATTAATGATAGAACCAGGGACACAAAATGTA
GGTGTTGTATTTTATGAATTATTCCTTGCATCTGGTTATCTCTTTCACGTACTTAACTGTT
TTTCCCACACTTATCACATTGTTCCTGCATACGGTTAATGGTGTAATTACATACTCCTCGT
TACCCAACATGCCGGGCATTCTTTTATATGCATAGGGTTCTCTTTTTTGGTTTCCCTTCAC
TTACATTTCAGAGTGCAGGCGCAACAAATAATTAAGGTTGTACTCTTCCCTTGGTCGCA
GTTAAAGTAGGTTAATTATTAAAAGACATAACTTAAGAATTACATATTAATAACTCAAGT
GCATAACATATCTATTCCTTCTTCAACTTACCCTTATATATATGCCCCCCTTTTGGTTTCTG
CGCGACAAACCCCCCTACCCCCTACGCTCAGCAAATCCTGTTATCCTTGTCAAACCCCG
AAACCAAGGAAGGTTCGAGAACGTGCAGACTAACAAGTTGGGATATGAATTAGCCATC
GCATTATATATATATACATGCATGCCGCGCTAGCCCATCGCATTAAAATCACCTAAAA

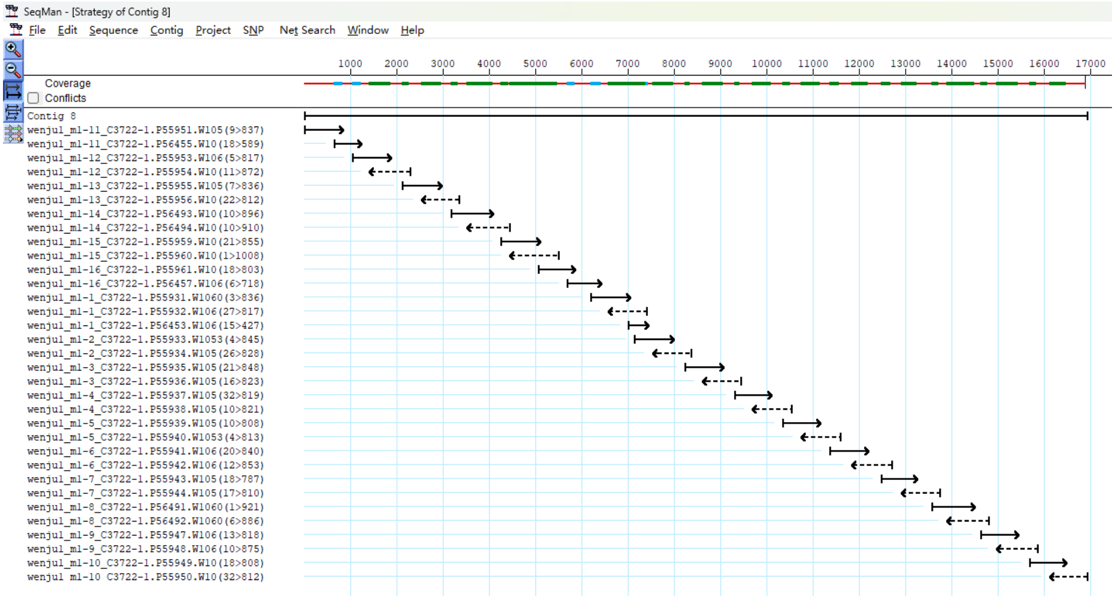

Supplementary Material S4. 16 pairs of primers sequencing assembly diagram which show the size and positions of overlaps. The strip below the ruler shows the number and coverage of the sequence. The color and thickness of the strips represent the number of overlay sequences. The green line indicates the area where the sequence overlaps. The blue lines indicate areas where only one strand has been sequenced. The red line indicates that this area has only been tested once.

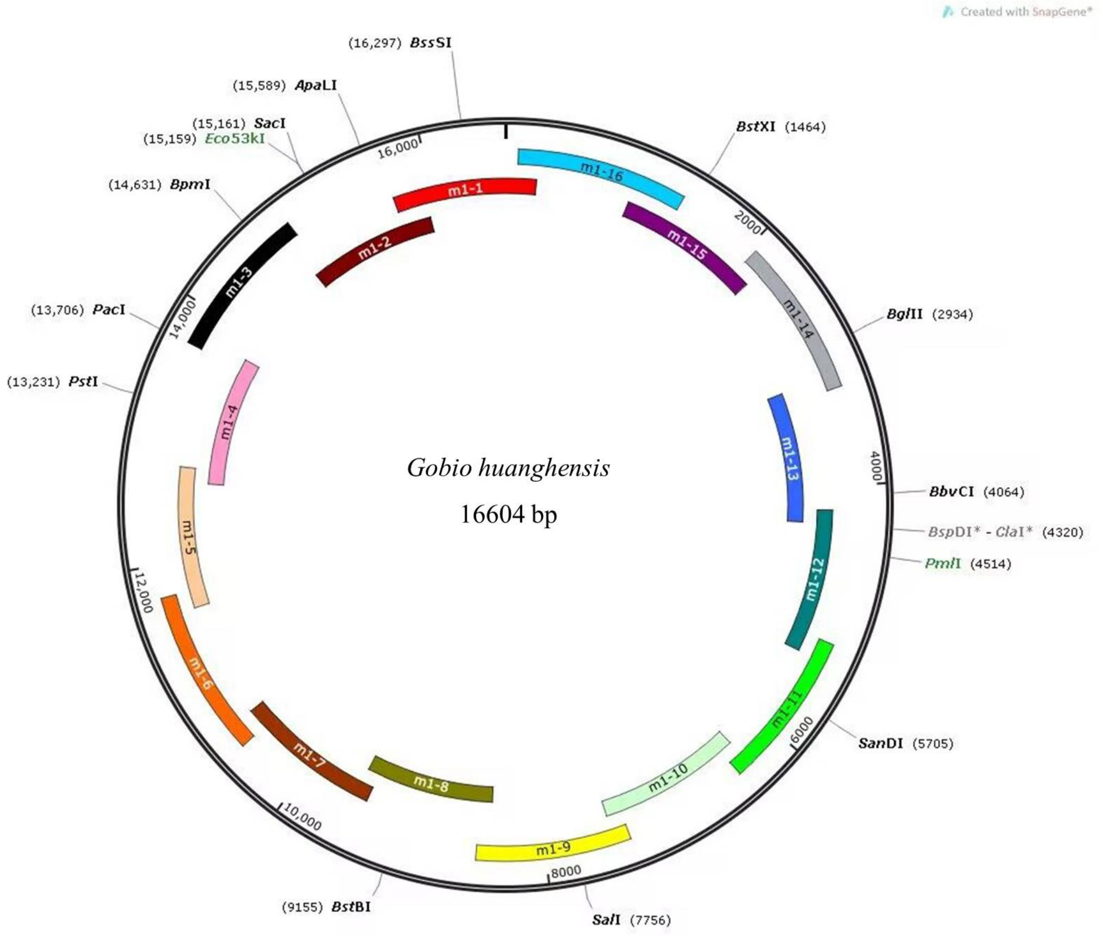

Supplementary Material S5. The mitochondrial genome map was maked using SnapGene, which shows the overlap between each fragment and forms a circular. They correspond to m1-1-m1-16 in Supplementary Material S1. The name outside the circle is the name of the restriction enzyme
